# Effect of arbuscular mycorrhizal fungi on the physiological functioning of maize under zinc-deficient soils

**DOI:** 10.1038/s41598-021-97742-1

**Published:** 2021-09-16

**Authors:** Abdul Saboor, Muhammad Arif Ali, Subhan Danish, Niaz Ahmed, Shah Fahad, Rahul Datta, Mohammad Javed Ansari, Omaima Nasif, Muhammad Habib ur Rahman, Bernard R. Glick

**Affiliations:** 1 Department of Soil Science, Faculty of Agricultural Sciences and Technology, Bahauddin Zakariya University, Multan, 60800 Pakistan; 2 Department of Agronomy, The University of Haripur, Haripur, 22620 Pakistan; 3 Department of Geology and Pedology, Faculty of Forestry and Wood Technology, Mendel University in Brno, Zemedelska1, 61300 Brno, Czech Republic; 4 Department of Botany, Hindu College Moradabad (Mahatma Jyotiba Phule Rohilkhand University Bareilly), Bareilly, 244001 India; 5 Department of Physiology, College of Medicine and King Khalid University Hospital, King Saud University, Medical City, PO Box-2925, Riyadh, 11461 Saudi Arabia; 6 Department of Agronomy, MNS-University of Agriculture-Multan, Multan, 60000 Pakistan; 7 Department of Biology, University of Waterloo, Waterloo, ON N2L 3G1 Canada

**Keywords:** Mass spectrometry, Plant sciences, Plant stress responses, Plant symbiosis

## Abstract

Zinc (Zn) deficiency can severely inhibit plant growth, yield, and enzymatic activities. Zn plays a vital role in various enzymatic activities in plants. Arbuscular mycorrhizal fungi (AMF) play a crucial role in improving the plant’s Zn nutrition and mitigating Zn stress effects on plants. The current study was conducted to compare the response of inoculated and non-inoculated maize (YH 1898) in the presence of different levels of zinc under greenhouse conditions under a Zn deficient condition. There were two mycorrhizal levels (i.e., M + with mycorrhizae, M- without mycorrhizae) and five Zn levels (i.e., 0, 1.5, 3, 6, and 12 mg kg^-1^), with three replicates following completely randomized design. At the vegetative stage (before tillering), biochemical, physiological, and agronomic attributes were measured. The results showed that maize plants previously inoculated with AMF had higher gaseous exchange traits, i.e., a higher stomatal conductance rate, favoring an increased photosynthetic rate. Improvement in antioxidant enzyme activity was also observed in inoculated compared to non-inoculated maize plants. Moreover, AMF inoculation also played a beneficial role in nutrients availability and its uptake by plants. Higher Zn12 (12 mg Zn kg^-1^ soil) treatment accumulated a higher Zn concentration in soil, root, and shoot in AMF-inoculated than in non-inoculated maize plants. These results are consistent with mycorrhizal symbiosis beneficial role for maize physiological functioning in Zn deficient soil conditions. Additionally, AMF inoculation mitigated the stress conditions and assisted nutrient uptake by maize.

## Introduction

Zinc (Zn) deficiency is widespread in arid and semi-arid regions, especially in alkaline calcareous soils, because of a high Zn fixation rate and Zn low mobility in such soil conditions^1,2^. Although a sufficient amount of Zn is present in the soil, only a small quantity of Zn is available for plant uptake. Zinc is mainly immobile in the soil as it is highly adsorbed to the surface of soil colloids^3^. Zinc deficiency is further aggravated by high soil pH, high soil Ca contents, mono-cropping, intensive agriculture and over the use of fertilizers. For instance, Rafique et al.^4^ reported that 80% of Pakistan's rainfed soil area is deficient in Zn due to high soil pH. Zinc deficiency of Zn in the surface layer of soils is widespread and its deficiency in plant tissues severely impacts their growth and reduces crop yields. Reduced plant concentrations of Zn stimulates reactive oxygen species (ROS) in plant tissues^5^. These ROS disrupt cell membranes and hinder normal cell functioning^6^. The ROS includes hydrogen peroxide and superoxide radicals, which cause extensive damage to plant tissue. On the other hand, Zn is involved in the gene expression, detoxification of ROS and activation of antioxidant enzyme production, including superoxide dismutase (SOD), peroxidase (PO) and catalase (CAT)^7^.

Most scientists suggest organic amendments, i.e., biochar^3,8–14^, biofertilizer^15–28^, humic substances, and organic matter^29–31^, to overcome the problem of abiotic and biotic stresses in crops. Arbuscular mycorrhizal fungi are well-known biofertilizers that protect host plants from biotic and abiotic stress by improving plant nutrition, especially P and Zn^8,9^. Extraradical hyphae of fungi explore the rhizosphere and bulk soil and enhance the uptake of nutrients with a low mobility rate like P, Zn and Cu. Most of the plants are colonized through AMF by Following two nutrients pathways. 1). Epidermis of roots, which is a direct pathway of uptake. 2). The formation of a fungal structure that designed mycorrhizal pathway uptake^10^.

Inoculation of mycorrhiza played an imperative role in the improvement of the chlorophyll contents of the plant. It also improved carotenoids contents end modified the synthesis of antioxidant enzymes, which regulates the metabolic activities under stress conditions^11^. Colonization of mycorrhiza increases the area under the rhizosphere through better branching of plant roots. Improvement in the branch also increases the uptake of water and nutrients, due to which growth and productivity of crops are increased^12–14^. Some studies have reported that mycorrhizal infection was sensitive to external P application, and thus, it affected the availability of other nutrients^15,16^. However, it was also reported that mycorrhizal fungi increased the uptake of Cu and contributed a 62% increase in Cu uptake irrespective of external P application^17^. Similarly, studies have highlighted the mycorrhizal fungi effect on Zn nutrition^7,18^. Therefore, instead of using inorganic sources for mitigating Zn deficiency, the use of mycorrhizal fungi as a bio-fertilizer may be an intriguing way of addressing Zn deficiency.

Subramanian et al.^7^ reported that mycorrhizal fungi trigger antioxidants that enable plants to survive in low Zn growing medium like Zn deficient soil. Studies have shown that mycorrhiza inoculated plants possess a high concentration of antioxidant enzymes above and below ground parts^19^. The AM fungi can increase the plant sugar and leaf proline contents and maintain plant cells' osmotic potential, protecting the plant’s enzymatic activities. AM fungi also increased the acid phosphatase activity in roots, leading to the improved nutritional value of inoculated plants^20^. Moreover, AM fungi also increased Zn and P concentrations in plant tissues, thereby positively affecting plant physiological processes such as photosynthesis, respiration, and stomatal conductance. Zinc is involved in chlorophyll synthesis; therefore, improved Zn nutrition in mycorrhizal plants stimulates chlorophyll synthesis^21^. So far, scientists have worked on AMF and Zn application solely under Zn deficiency to improve growth and yield in maize. However, the current study will cover the knowledge gap with the novel aspect of AMF and Zn application rates influence on antioxidants and physiological attributes of maize under Zn deficient. That’s why the current study was conducted to explore the efficacy of AMF for better uptake of Zn in maize in Zn deficient soil. It is hypothesized that AMF has the potential to mitigate Zn deficiency by unbalanced Zn application in maize.

## Materials and methods

### Experimental soil and location

The soil from the experimental site was aridisol, clay loam in texture, alkaline in pH (8.3), and contained low organic matter (0.58%)^22^. The available phosphorus level was 6.4 mg/kg extracted with NaHCO_3_^23^, the potassium level was 225 mg/kg^24^, and the Zn level was 0.45 mg/kg^25^. The soil was taken from field latitude 29.92 and longitude 71.52.

### Experimental site and design

A pot experiment was conducted in loamy soil to evaluate Zn deficiency effects on a maize crop's physiological functioning at the research area of the Department of Soil Science Bahauddin Zakaria University, Multan Pakistan. The study was arranged according to a completely randomized design (CRD).

### Pot preparation and treatments

For this purpose, clay pots with 10 kg soil were filled with a mixture of soil and sand in a 4:1 ratio; the purpose of adding sand is to provide a mixture from which roots can be easily extracted. Plants were grown for a total period of 45 days after sowing. The treatments were two mycorrhizal levels (i.e., M^+^ with mycorrhizae, M^-^ without mycorrhizae) and five Zn levels (i.e., 0, 1.5, 3, 6, and 12 mg/kg), with the entire experiment being repeated three times. The required quantity of Zn was applied using analytical grade zinc heptahydrate (ZnSO_4_·7 H_2_O).

### Fertilizer application and AMF inoculation

Nitrogen fertilizer (227.24 kg ha^-1^) was applied in three separate aliquots. Half of P (71.63 kg ha^-1^) and full recommended dose of K (91.93 kg ha^-1^) were applied as a basal dose at the time of sowing. The AM inoculum containing a consortium of *Glomus* species, i.e., mycorrhizal inoculum Clonex® (Root Maximizer; 5711 Enterprise Drive, Lansing, MI, USA) having 158 propagules gram^-1^ was applied at 25 g inoculum per pot. Thiophanate methyl fungicide was applied to the control treatments at 50 mg kg^-1^ fortnight^-1^ pot^-1^ to control the root mycorrhizal colonization.

### Seeds sowing and irrigation

Five maize seeds (YH 1898 cv.) were sown per pot. After 20 days of growth, only 2 healthy plants per pot were maintained. The selection was made based on visual observations. The moisture content in each pot was maintained at 60% of the total WHC.

### Harvesting

At the vegetative stage (before tillering), biochemical, physiological, and agronomic attributes were measured.

#### Tissue and soil analysis

Fresh plant leaves and roots were excised, rinsed with water and oven-dried at 72ºC for 24 h. Plant leaf and fresh root weights and dried weights were recorded. Total N was determined by the calorimetric method^26^. Phosphorus was analyzed using the malachite green method^27^. Potassium was determined by flame photometer^28^. Pre- and post-treatment soil samples were collected to observe the nutrient status of the soil. Soil zinc was extracted by the DTPA extraction method and analyzed using an Atomic Absorption Spectrophotometer (AAS)^25^. The soil pH^29^ and EC^30^ were determined in soil water suspensions of 1:5 (soil:water) ratios using pH and EC meters.

#### Physiological parameter

The stomatal conductance, photosynthetic rate, and transpiration rate were measured using an IRGA (infrared gas analyzer). All physiological parameters were measured between 9 and 11 AM to avoid low humidity and high external temperature^31^.

#### Chlorophyll contents

Chlorophyll contents were determined by the procedure of Arnon^32^. The intensity of green color extract from fresh plant leaves was measured at 645 and 663 nm^32^.

$$\mathrm{Chlorophyll\, a }\left(\mathrm{mg}\,{\mathrm{g}}^{-1} Fresh\, Weight\right)=\frac{12.7 \left(\mathrm{value\, at\, }663\,\mathrm{nm}\right)-2.69 (\mathrm{value\, at\, }645\,\mathrm{nm})}{1000\, (\mathrm{W})}$$

$$\mathrm{Chlorophyll\, b }\,\left(\mathrm{mg}\,{\mathrm{g}}^{-1} Fresh\, Weight\right)=\frac{22.9 \left(\mathrm{value\, at\, }645\,\mathrm{nm}\right)-4.68 (\mathrm{value\, at\, }663\,\mathrm{nm})}{1000 (\mathrm{W})}$$

$$\mathrm{Total \,Chlorophyll }\left(\mathrm{mg}\,{\mathrm{g}}^{-1} Fresh \,Weight\right)=\frac{20.2 \left(\mathrm{value\, at\, }645\,\mathrm{nm}\right)-8.02 (\mathrm{value\, at\, }663\,\mathrm{nm})}{1000 (\mathrm{W})}$$

*W = Fresh weight of leaves.

#### Enzymatic analysis

Determination of antioxidant enzymes was done by collecting fresh leaves, which were immediately kept in liquid nitrogen. Later, 1 g of sample was ground and completely homogenized in 50 mM phosphate.

#### Superoxide dismutase

This enzyme was measured by the method of Giannopolitis and Ries^33^. A mixture of 1 mL nitroblue tetrazolium (NBT) (50 mM), 500 μL ethylenediaminetetraacetic acid (EDTA) (75 mM), 1 mL riboflavin (1.3 μM), 950 μL sodium phosphate buffer (50 mM), and 0.5-mL methionine (13 mM) was prepared. A 50 μL sample extract was added, and the mixture was placed under fluorescent light for 5 min. The absorbance of the resultant solution was measured at 560 nm. The SOD activity was measured in IU min^−1^ mg^−1^ protein.

#### Catalase

A mixture of 0.9 mL H_2_O_2_ (5.9 mM) in 2 mL phosphate buffer (50 mM). was used to measure the catalase activity (CAT). 0.1 mL of an enzyme extract was mixed with the mixture. After 30 s the absorbance was measured at 240 nm and monitored continuously for 5 min to measure the disintegration of H_2_O_2_ by CAT activity^34^. The CAT activity was measured in μmol min^−1^ mg^−1^ protein.

#### Peroxidase

100 μL enzyme extract was added to a solution of 2 mL sodium phosphate buffer (50 mM), 400-μL guaiacol (20 mM), and 500 μL H_2_O_2_ (40 mM). The absorbance of this mixture was measured at an interval of 20 s for 5 min. The peroxidase (POD) concentration was calculated by the absorbance change^34^. The POD activity was measured in μmol min^−1^ mg^−1^ protein.

#### Total soluble proteins (TSP)

The method of Sambrook and Russell^35^ was used to determine the total soluble proteins, and the Bradford^36^ method was used to estimate the TSP. A 200 μL of either leaf or root extract was mixed into 780 μL DI water and 20 μL of Coomassie blue dye. The absorbance of this sample was measured at 595 nm. The TSP was expressed in mg mL^−1^.

### Mycorrhizal colonization

The fine roots of maize plants were harvested and then washed with KOH (10% w/v)^37^. The root was then stained with a 0.05 trypan blue solution^38^. The gridline intersects method was used to search for the presence of AM structure in roots.

### Statistical analysis

The data was analyzed using two factorial analysis of variance (ANOVA) and treatments were compared using the Least Significant Difference (LSD) test at *p* ≤ 0.05^39^ on Statistical Package Statistix 8.1 (Tallahassee Florida, USA). Correlation and principal component analysis (PCA) were performed using XLSTAT-2014.

### Institutional, national, and international guidelines and legislation

Current experiment meets all international, national and/or institutional guidelines for experimental research and field studies on plants (either cultivated or wild), including the collection of plant material.

### Relevant permits/permissions/licences

For collection of maize seeds all relevant permits or permissions have been obtained.

### Ethics approval and consent to participate

We all declare that manuscripts reporting studies do not involve any human participants, human data, or human tissue. So, it is not applicable.

### Complies with international, national and/or institutional guidelines

Current experiment meets all international, national and/or institutional guidelines.

## Results

### Colonization of mycorrhizal fungi

**Figure 1:**
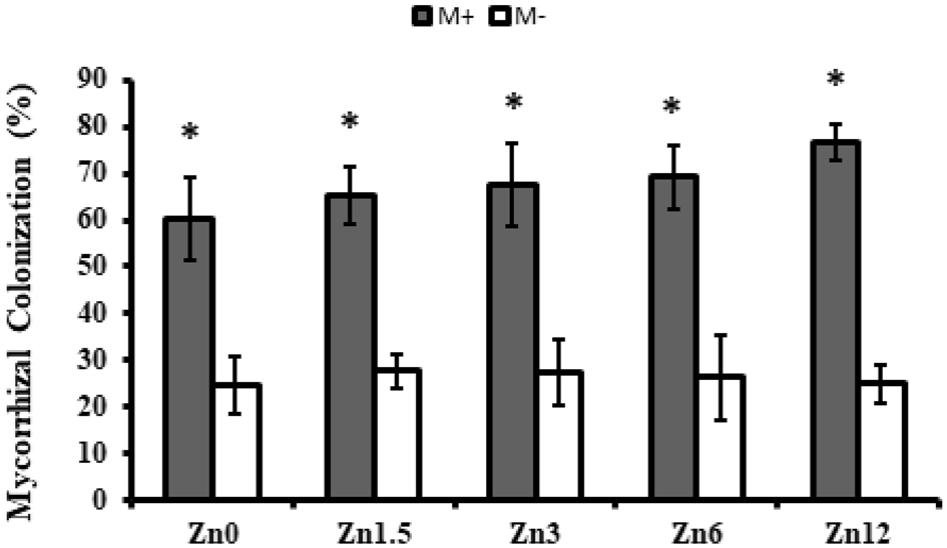
Effect of arbuscular mycorrhizal fungi inoculation on mycorrhizal colonization by maize under zinc deficient soil conditions Zn_0_ (control), Zn_1.5_ (1.5 mg kg^-1^_),_ Zn_3_ (3 mg kg^-1^), Zn_6_ (6 mg kg^-1^), Zn_12_ (12 mg kg^-1^), inoculated with AMF (M + grey), un-inoculated (M- white). Error bars represent standard error. Inoculated with AMF (M + grey); un-inoculated (M- white).

Data regarding mycorrhizal colonization was measured from the roots of both inoculated and un-inoculated maize plants. A significant (*p* ≤ 0.05) increase in mycorrhizal colonization was observed following inoculation irrespective of zinc levels. The inoculated roots showed colonization of mycorrhiza in the range of 60–76% whereas, un-inoculated roots had 24–27% root colonization. Maximum colonization was observed with the treatment of Zn12 as compared to the other treatments (Fig. 1).

### Influence of AMF on gaseous exchange traits

**Figure 2:**
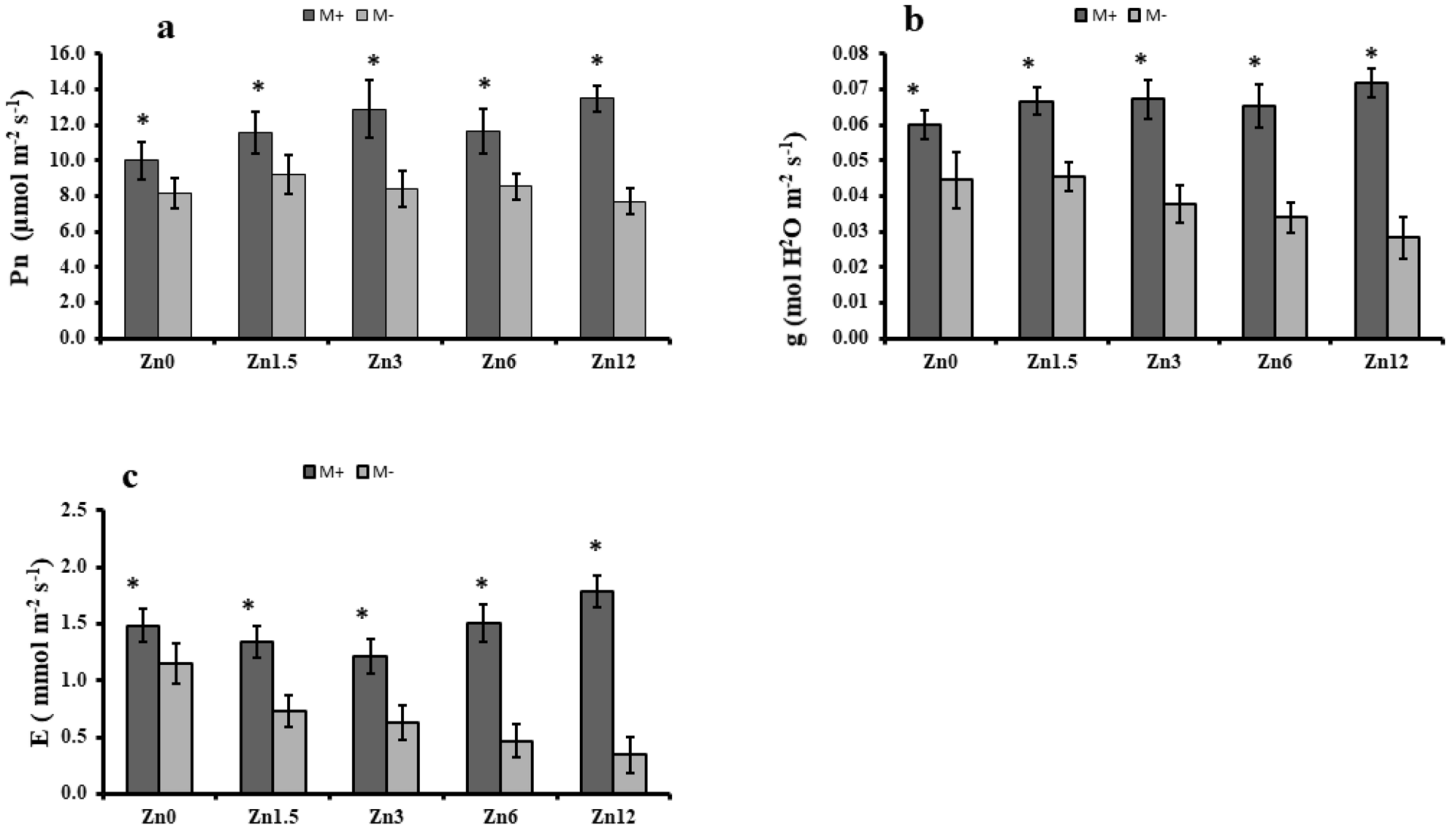
Effect of arbuscular mycorrhizal inoculation on gaseous exchange traits of maize under zinc deficient soil conditions. (**a**) Photosynthetic rate (µmol m^-2^ s^-1^) (**b**) stomatal conductance (mol H_2_O m^-2^ s^-1^) (**c**) transpiration rate (mmol m^-2^ s^-1^). Zn_0_ (control), Zn_1.5_ (1.5 mg kg^-1^_),_ Zn_3_ (3 mg kg^-1^), Zn_6_ (6 mg kg^-1^), Zn_12_ (12 mg kg^-1^), inoculated with AMF (M + grey), un-inoculated (M- white). Error bars represent standard error and asterisk (*) shows a significant difference (*p* ≤ 0.05) among treatments (Zn × AMF interaction).

The stomatal conductance rate consistently increased with the increment in the zinc fertilization levels. A significant (*p* ≤ 0.05) difference was noticed among inoculated and non-inoculated maize plants. The highest stomatal conductance rate was measured from Zn12 inoculated with an increase of 12% compared to the control, whereas its counterpart showed the least stomatal conductance rate with the decrease of 36% in M- compared to the control (Fig. 2). Changes to the photosynthetic rate were similar to those observed for the stomatal conductance rate. The Zn12 (M +) showed the highest photosynthetic rate with an increase of 35%, while M- had a 6% decrease in photosynthetic rate. The remaining inoculated plants also had a higher photosynthetic rate at different zinc levels, showing significant (*p* ≤ 0.05) differences compared to non-inoculated plants. The M + plant with the highest zinc level (Zn12) tested in this study exhibited a higher transpiration rate with a 56% increase compared to the control (Zn0), while M- plants with Zn12 treatment exhibited a 77% decrease in transpiration rate compared to the Zn0 (Fig. 2).

### Arbuscular mycorrhizal fungi effect on chlorophyll contents

**Figure 3:**
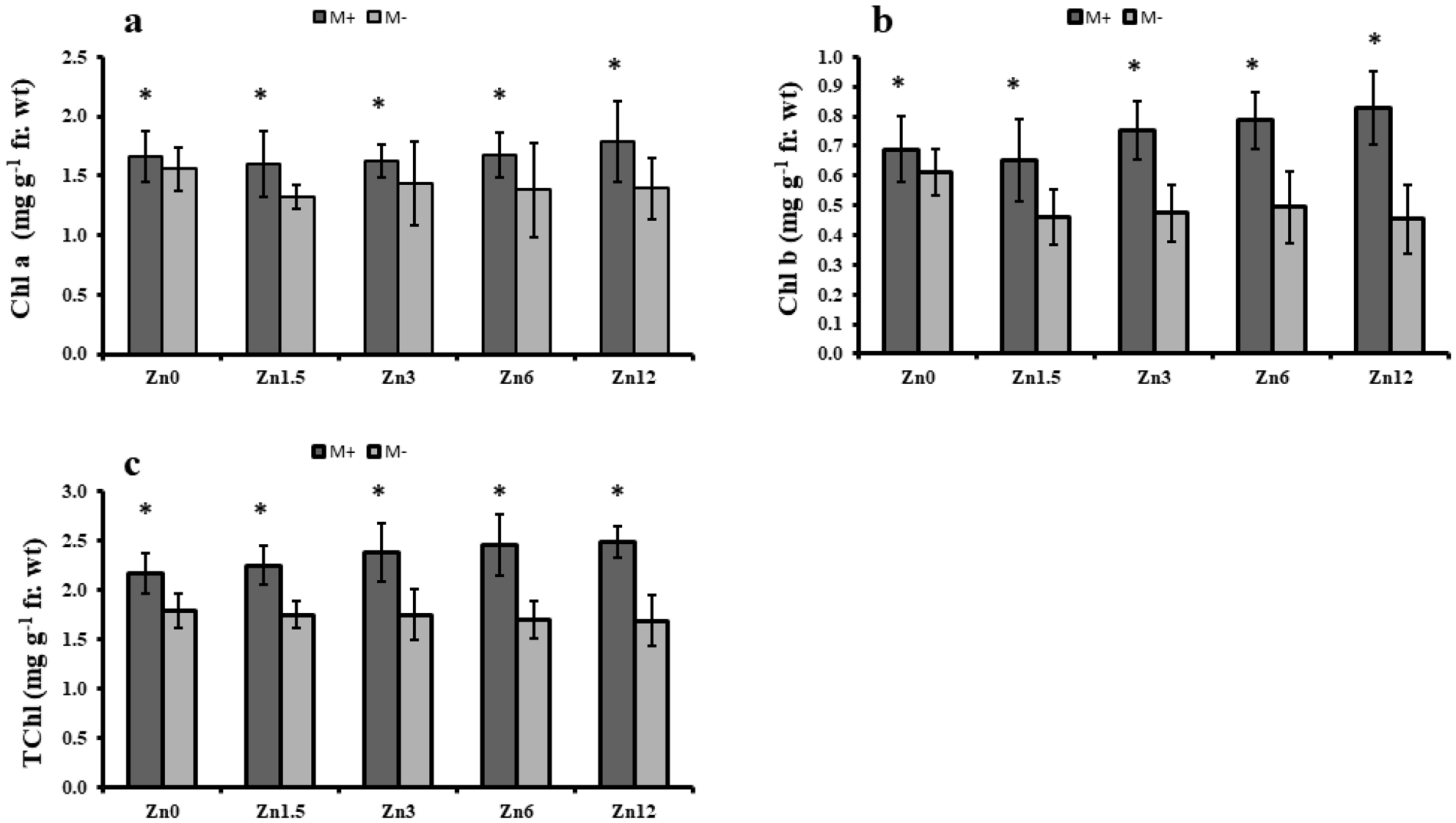
Effect of arbuscular mycorrhizal inoculation on chlorophyll contents of maize under zinc deficient soil conditions. (**a**) chlorophyll a content (mg g^-1^ fr. wt) (**b**) chlorophyll b contents (mg g^-1^ fr. wt) (**c**) total chlorophyll contents (mg g^-1^ fr. wt). Zn_0_ (control), Zn_1.5_ (1.5 mg kg^-1^_),_ Zn_3_ (3 mg kg^-1^), Zn_6_ (6 mg kg^-1^), Zn_12_ (12 mg kg^-1^), inoculated with AMF (M + grey), un-inoculated (M- white). Error bars represent standard error and asterisk (*) shows a significant difference (*p* ≤ 0.05) among treatments (Zn × AMF interaction).

This study also quantified the change in chlorophyll contents with the varying zinc levels with and without mycorrhizal inoculation; these values tend to increase more with mycorrhizal inoculation. All the chlorophyll contents (a, b, and total) indicated an increase in inoculated (M +) plants compared to M- plants. The chlorophyll a content was found to increase by 8% in Zn12, although a decrease of up to 10% was observed in non-inoculated plants. Likewise, the chlorophyll b content increased by 20% (Zn12) in M + plants and showed a 26% decrease in non-inoculated plants. Similar results were observed for total chlorophyll contents in that Zn12 (M +) had a 15% higher total chlorophyll contents, while Zn (M-) resulted in a decline of 6% (Fig. 3).

### Antioxidant enzymes activity

**Figure 4:**
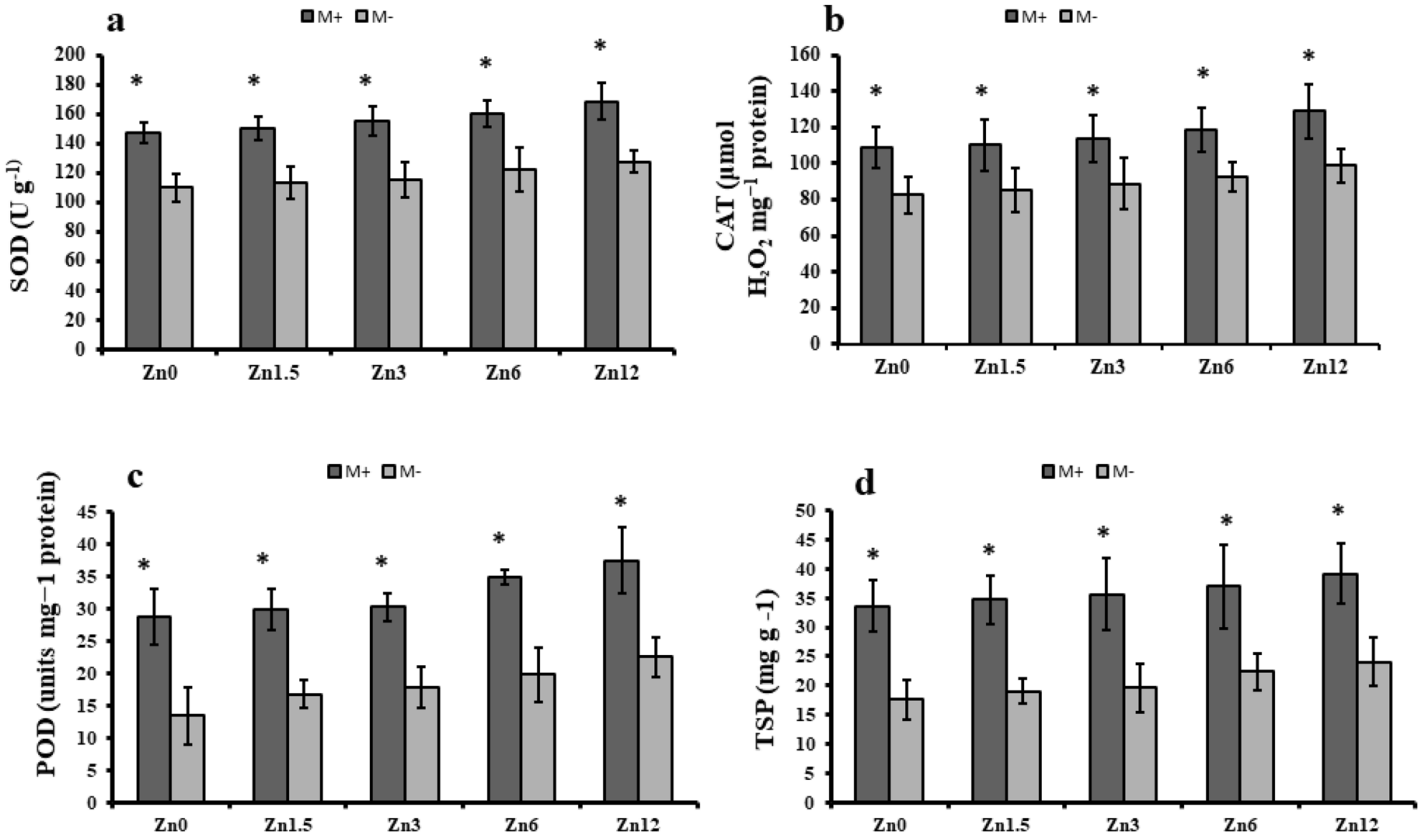
Effect of arbuscular mycorrhizal inoculation on antioxidant enzyme activity of maize under zinc deficient soil conditions. (**a**) superoxide dismutase (U g^-1^) (**b**) catalase (μmol H_2_O_2_ mg^−1^ protein) (**c**) peroxidase (U mg^−1^ protein), (**d**) total soluble protein (mg g ^-1^). Zn_0_ (control), Zn_1.5_ (1.5 mg kg^-1^_),_ Zn_3_ (3 mg kg^-1^), Zn_6_ (6 mg kg^-1^), Zn_12_ (12 mg kg^-1^), inoculated with AMF (M + grey), un-inoculated (M- white). Error bars represent standard error and asterisk (*) shows a significant difference (*p* ≤ 0.05) among treatments (Zn × AMF interaction).

Mycorrhizal inoculated plants showed significantly (*p* ≤ 0.05) higher enzymatic activities than non-inoculated plants at each Zn level. The highest superoxide dismutase activity was observed in M + at Zn12 with an increase of 15%; M- also showed higher SOD activity by 16% compared to the control. The catalase activity was also higher with the higher level of zinc fertilization (Zn12). It showed a 19% increase in M + and a 20% increase in M- compared to their respective controls. Peroxidase also exhibited elevated enzyme activity. It was 30% higher in Zn12 with M + and 68% higher in M than their respective controls (Fig. 4). Mycorrhizal inoculation and Zn fertilization resulted in a significant (*p* ≤ 0.05) increase in maize plants' total soluble protein; these levels increased gradually with the increase in Zn levels regardless of M + and M- treatments. The highest TSP was recorded from the Zn12 in M + with a 17% increase whereas, M- showed a 36% increase in TSP (Fig. 4).

### Zinc concentration of the soil

**Figure 5:**
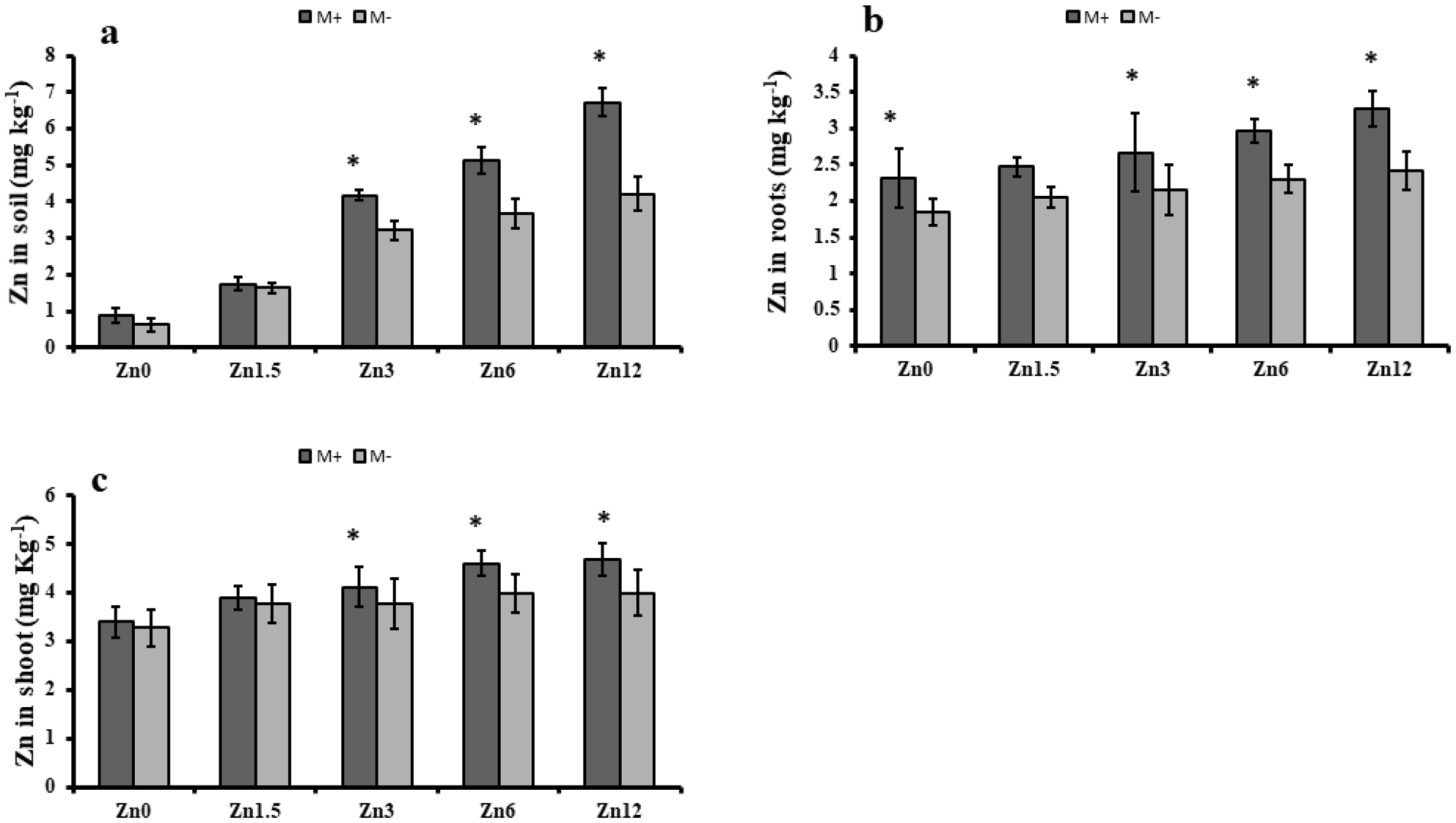
Effect of arbuscular mycorrhizal inoculation on zinc concentration of maize under zinc deficient soil conditions. (**a**) zinc concentration in soil (mg kg^−1^) (**b**) zinc concentration in root (mg kg ^−1^) (**c**) zinc concentration in shoot (mg kg ^−1^). Zn_0_ (control), Zn_1.5_ (1.5 mg kg^-1^_),_ Zn_3_ (3 mg kg^-1^), Zn_6_ (6 mg kg^-1^), Zn_12_ (12 mg kg^-1^), inoculated with AMF (M + grey), un-inoculated (M- white). Error bars represent standard error and asterisk (*) shows significant difference (*p* ≤ 0.05) among treatments (Zn × AMF interaction).

The zinc concentration of zinc-deficient soil increased with the addition of zinc with mycorrhizal inoculation. The highest Zn accumulation from Zn12, increased by 41%; the lowest Zn accumulation was measured from Zn0 (control) with mycorrhizal inoculation. In non-mycorrhizal inoculation, the roots accumulation was also significantly higher under Zn12 compared to control maize plants. Zn12 also resulted in a higher uptake of the Zn in shoots, i.e., a 38% increase in shoots compared to the control. Similarly, with non-inoculated plants, Zn accumulation was 22% higher in shoots (Fig. 5).

### Phosphorus availability and uptake by maize

**Figure 6:**
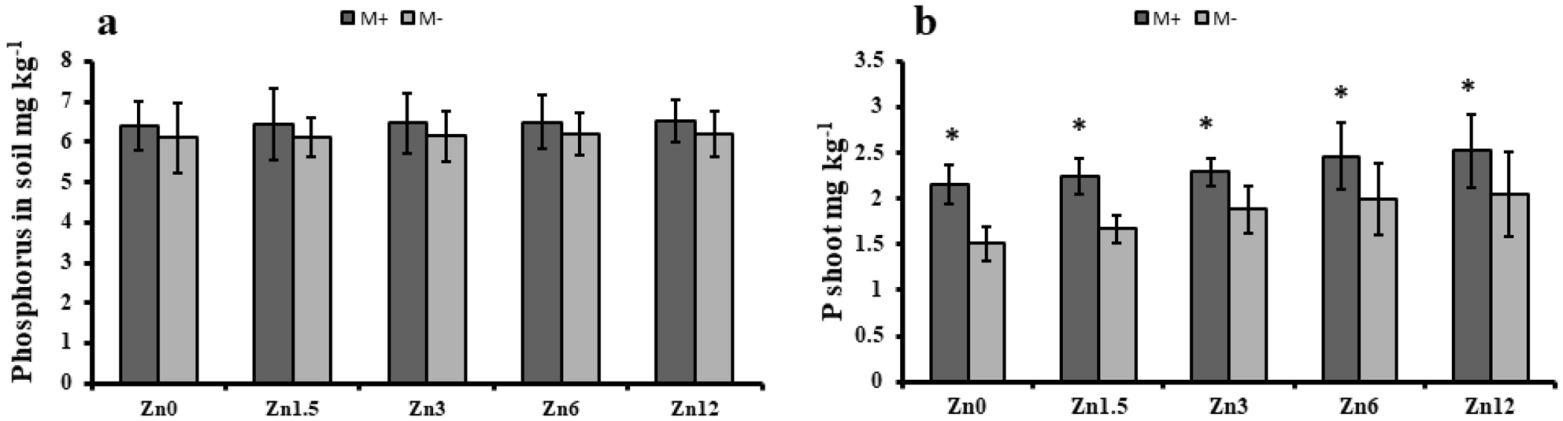
Effect of arbuscular mycorrhizal inoculation on Phosphorus contents of maize under zinc deficient soil conditions. (**a**) phosphorus concentration in soil (mg kg^−1^) (b) phosphorus concentration shoot (mg kg ^−1^). Zn_0_ (control), Zn_1.5_ (1.5 mg Kg^-1^_),_ Zn_3_ (3 mg kg^-1^), Zn_6_ (6 mg kg^-1^), Zn_12_ (12 mg kg^-1^), inoculated with AMF (M + grey), un-inoculated (M- white). Error bars represent standard error and asterisk (*) shows significant difference (*p* ≤ 0.05) among treatments (Zn × AMF interaction).

In the case of phosphorus availability in soil, Zn12 plants only showed a 2% increase in phosphorus accumulation in soil, whereas under M- plants exhibited a 6% increase compared to the control. Moreover, the phosphorus accumulation in shoots was also higher under M- conditions and Zn12, while M + showed 17% increase in shoot phosphorus levels. Finally, the higher application of zinc (Zn12) and mycorrhizal inoculation resulted in higher zinc availability in maize soil (Fig. 6).

### Correlation analysis

The Pearson correlation coefficients (r) for the different physiological and biochemical parameters under zinc-deficient soil conditions are summarized in Table 1. All of the parameters showed a positive correlation. The photosynthetic rate showed a strong positive correlation with stomatal conductance, superoxide dismutase, total soluble protein, total chlorophyll, zinc, and phosphorus in soil. The antioxidant enzymes (SOD, CAT, POD) showed a strong positive correlation with zinc accumulation in the root, shoot and soil, and phosphorus accumulation in soil and maize shoots. Also, these antioxidant enzymes were positively correlated with the maize chlorophyll contents.

**Table 1:** Pearson’s correlation coefficients between the variables under zinc-deficient soil conditions.

	Photo	Trans	Cond	SOD	CAT	POD	TSP	CHLA	CHLB	TCHL	ZR	ZSH	ZS	PS	PSH
Photo	1	0.65	0.95**	0.81*	0.752	0.64	0.81*	0.46	0.69	0.80*	0.77	0.777	0.84*	0.80*	0.712
Trans		1	0.78	0.92*	0.94**	0.96**	0.94**	0.80*	0.75	0.80	0.93*	0.86*	0.84*	0.93*	0.92*
Cond			1	0.82*	0.79	0.70	0.84*	0.50	0.60	0.76	0.79	0.78	0.79	0.85*	0.74
SOD				1	0.98**	0.96**	0.99**	0.82*	0.92*	0.93**	0.99**	0.93*	0.97**	0.99**	0.96**
CAT					1	0.96**	0.98**	0.88*	0.90*	0.87*	0.98**	0.88*	0.93**	0.98**	0.94**
POD						1	0.96**	0.82*	0.88*	0.89*	0.98**	0.92*	0.92*	0.96**	0.98**
TSP							1	0.79	0.89*	0.93**	0.99**	0.94**	0.97**	0.99**	0.97**
CHLA								1	0.80	0.60	0.79	0.58	0.71	0.79	0.73
CHLB									1	0.91*	0.92*	0.85*	0.95**	0.90*	0.89*
TCHL										1	0.95**	0.98**	0.98**	0.94**	0.95**
ZR											1	0.95**	0.97**	0.99**	0.98**
ZSH												1	0.95**	0.94**	0.97**
ZS													1	0.97**	0.95**
PS														1	0.96**
PSH															1

**Highly significant (*p* < 0.01), *significant (*p* < 0.05). Photo, photosynthetic rate; Trans, transpiration rate; Cond, stomatal conductance; SOD, superoxide dismutase; CAT, catalase; POD, peroxidase; TSP, total soluble protein; CHLA, chlorophyll a; CHLB, chlorophyll b; TCHL, total chlorophyll; ZR, zinc in roots; ZSH, zinc in shoot; ZS, zinc in soil; PS, phosphorus in soil; PSH, phosphorus in shoot.

### Principal component analysis

**Figure 7:**
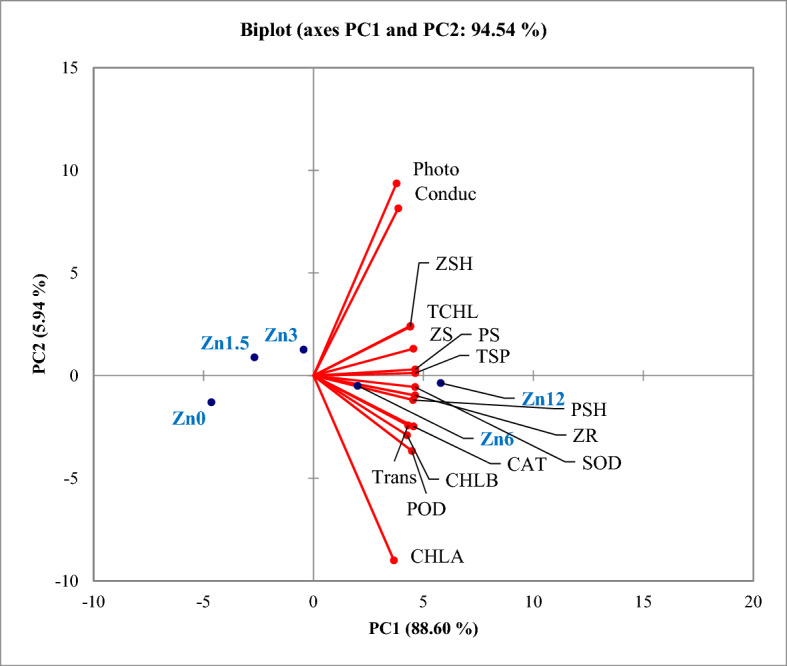
PCA biplot for different zinc levels under zinc deficient soil conditions. PCA biplot is a combination of score plot of zinc levels (represented in blue text) and loading plot of variables (represented by red vectors; black text). Photo, photosynthetic rate; Trans, transpiration rate; Conduc, stomatal conductance; SOD, superoxide dismutase; CAT, catalase; POD, peroxidase; TSP, total soluble protein; CHLA, chlorophyll a; CHLB, chlorophyll b; TCHL, total chlorophyll; ZR, zinc in roots; ZSH, zinc in shoot; ZS, zinc in soil; PS, phosphorus in soil; PSH, phosphorus in shoot.

The interrelationship among selected zinc levels on zinc-deficient soil both with and without mycorrhizal inoculation were analysed by biplot principal component analysis (PCA), as shown in Fig. 7. This plot revealed that the first two components explained 94.54% of the variation (PC1 88.60%, and PC2 5.94%). The PCA biplot grouped the zinc levels based on maize performance observed for physiological and biochemical variables, showing that the Zn12 was the best performing situation for improving the maize's overall traits in this experiment. The Zn12 treatment also helped to improve the nutrient status of the soil. The Zn6 treatment also provided significant improvement in the measured characteristics. However, the Zn0, Zn1.5 and Zn3 treatments had a much smaller effect in significantly improving maize growth under zinc deficient soil conditions.

## Discussion

The current study has elaborated the beneficial role of AM colonization compared to non-mycorrhizal plants. Arbuscular mycorrhizal symbiosis alters plants' internal physiological conditions, consequently controlling the opening of stomata, one of the prime sites of photosynthesis^40,41^. A higher photosynthetic rate was attributed to the Zn12 (M +) condition compared to non-mycorrhizal plants in this study. Another study supported this result in which it was reported that regulation of abscisic acid and antioxidant enzyme activities are correlated with each other in altering stomatal behaviour^42^. Our results highlight AM symbiosis' importance that can increase the chlorophyll contents of maize in nutrient-deficient soils. Furthermore, mycorrhizal inoculation might enhance the magnesium uptake, which helps develop higher chlorophyll contents similar to what was observed in Zn12, ultimately resulting in a higher photosynthetic rate^43^. These findings agree with earlier studies' results^44,45^. Presence of AMF protects photosystem II during the photosynthesis that played a significant role in better uptake of nutrients and water. Balance uptake of water Regulate photosynthetic rate through improvement in transpiration rate in stomatal conductance. On the other hand, optimum uptake of zinc properly catalyzed the reactions Which facilitate the plants for better growth^46–49^.

Poor uptake of Zn facilitates the biosynthesis of reactive oxygen species (ROS) during photosynthetic electron transport^50^. However, antioxidants play a critical role in defense system of cells components such as proteins, membrane lipids, SH-containing enzymes, chlorophyll and DNA against ROS. The cysteine, histidine and glutamate or aspartate residues represent the most critical Zn binding sites in enzymes, DNA-binding proteins (Zn-®nger proteins) and membrane proteins^6^. Improvement in antioxidant enzyme activities were also observed in maize tissue samples of current study (Fig. 4). Since SOD is the main ROS scavenging enzyme that converts superoxide ion to H_2_O_2_ (which is also harmful to plant growth), the activity CAT and POD was also shown to be increased in our maize plants. In the present study, plants were exposed to artificial Zn deficient soil conditions, that might result in ROS species' overproduction. However, AM-inoculated maize plants produced higher antioxidant enzyme activity, favouring ROS stress molecule scavenging which diminished the oxidative burst^6^. Both enzyme activities were significantly (*p* ≤ 0.05) higher with AM inoculation than non-inoculated plants. It might be the reason that inoculated plants showed higher SOD activity in this study. Moreover, this enzyme activity also contributed to hyphal transport, which assists in diffusing essential micronutrients necessary for enzyme activities^7^. The AM role observed was supported by an earlier report that the vegetative part (mycelium) of mycorrhizal fungi in the rhizosphere enhance the availability of nutrients to roots.

The maize plant’s total soluble protein contents increased in inoculated and non-inoculated plants, although the increased level was highest with inoculated plants with a Zn dose of 6 mg kg^-1^ of soil. Our results regarding the increase in soluble proteins agree with previous reports^7^. In a separate study, mycorrhizal inoculation resulted in a soluble protein increase of 2–sixfold in clover plants^51^.

The higher availability of Zn in the soil might be due to the AM role in synthesizing amino acids (tryptophan and asparagine). Therefore, the current study showed that higher levels of Zn resulted in higher Zn availability in soil. Likewise, it allowed the plants a higher level of Zn uptake. Our results regarding higher zinc accumulation were consistent with the results of the earlier report of Marques et al.^52^ and are also confirmed by another study on maize, concluding that mycorrhizal inoculation renders a positive role in the accumulation of Zn under zinc-deficient soil^53^.

Phosphorus is a relatively immobile nutrient in soil yet its concentration in the soil increased with the elevated levels of Zn fertilization irrespective of mycorrhizal inoculation. However, in the case of Zn uptake in shoots, contrary results showed by inoculated maize plants. It might be the reason that M + assisted maize plants in the absorption of phosphorus by the roots. Numerous studies have reported AM positive role in phosphorus accumulation^28,54^. Moreover, increasing the level of Zn increased the root surface area of the plant with mycorrhizal colonization. It also secreted the glomalin protein that solubilizes the fixed phosphorus, resulting in a higher concentration of phosphorus in the soil.

## Conclusions

The current study showed the beneficial role of arbuscular mycorrhizal inoculation for maize growth on zinc-deficient soil. It improved the maize's gaseous exchange traits, such as a higher photosynthetic rate and a higher zinc application level. The chlorophyll contents also improved with higher zinc fertilization and inoculation of maize plants. These favourable maize physiology changes were solely due to a higher nutrient availability under deficient soil conditions. Inoculated plants also showed a higher accumulation of nutrients and their availability in soil.
